# RCPred: RNA complex prediction as a constrained maximum weight clique problem

**DOI:** 10.1186/s12859-019-2648-1

**Published:** 2019-03-29

**Authors:** Audrey Legendre, Eric Angel, Fariza Tahi

**Affiliations:** IBISC, Univ Evry, Université Paris-Saclay, Evry, 91025 France

**Keywords:** RNA complex, Secondary structure, RNA interaction, Pseudoknot, Maximum weight clique heuristic

## Abstract

**Background:**

RNAs can interact and form complexes, which have various biological roles. The secondary structure prediction of those complexes is a first step towards the identification of their 3D structure. We propose an original approach that takes advantage of the high number of RNA secondary structure and RNA-RNA interaction prediction tools. We formulate the problem of RNA complex prediction as the determination of the best combination (according to the free energy) of predicted RNA secondary structures and RNA-RNA interactions.

**Results:**

We model those predicted structures and interactions as a graph in order to have a combinatorial optimization problem that is a constrained maximum weight clique problem. We propose an heuristic based on Breakout Local Search to solve this problem and a tool, called RCPred, that returns several solutions, including motifs like internal and external pseudoknots. On a large number of complexes, RCPred gives competitive results compared to the methods of the state of the art.

**Conclusions:**

We propose in this paper a method called RCPred for the prediction of several secondary structures of RNA complexes, including internal and external pseudoknots. As further works we will propose an improved computation of the global energy and the insertion of 3D motifs in the RNA complexes.

**Electronic supplementary material:**

The online version of this article (10.1186/s12859-019-2648-1) contains supplementary material, which is available to authorized users.

## Background

RNAs can link to each other and form complexes having catalytic functions. A well known example is the ribosome [1] composed of the 5S, 5.8S, 18S and 28S RNAs (in eucaryotes) and of ribosomal proteins. The RNAs present in the ribosome are involved in the formation of peptid bonds and in the reading of codons in the site A. RNA complexes are formed by canonical interactions (the Watson-Crick base pairs (A-U, G-C) and the Wobble base pair (G-U)) between several RNA strands and by non canonical interactions (tertiary interactions). Canonical interactions are the strongest interactions that stabilize RNA structure and they define the secondary structure. Non-canonical interactions are weaker but more numerous than canonical interactions. They involve chemical H bonds in the Hoogsteen or the sugar edges of the nucleotides and are responsible of the RNA 3D structure. In this paper, we focus only on the strongest interactions involved in RNA complexes, i.e. the canonical interactions.

Many tools exist to predict the joint secondary structure of RNA duplexes (both the base pairs internal to each RNA and the interaction or hybridization base pairs) using either the thermodynamic approach [2–9] or the comparative approach [6, 10–12]. However, to predict the secondary structure of RNA complexes composed of more than two RNAs is difficult and very few dedicated tools exist. The first proposed tool was MultiRNAFold [13]. In this tool, the RNAs are connected as one strand with extra loops between them. The minimum free energy of the complex is computed by a dynamic programming algorithm derived from [14]. The NUPACK package [15], proposed later, includes a software to predict RNA complex secondary structures. It extends the partition function computation of a single RNA, allowing the computation of the minimum free energy structure, to the multiple RNA case. Later, the RNA complex secondary structure prediction is modeled as a multiple RNA interaction problem [16]. The authors proved that this problem is NP-hard and proposed several approximation algorithms. Then, the RNA complex secondary structure prediction is formulated as a combinatorial optimization problem called *Pegs and Rubber Bands* and an approximation algorithm is proposed [17]. Then the tools NanoFolder [18] HyperFold [19] for RNA complex prediction are proposed. NanoFolder works in two steps, first it computes all the possible helices using a simple energy model and then a greedy algorithm selects the minimum free energy helices and adds them into the RNA complex. HyperFold also generates the possible helices but uses a more sophisticated algorithm than NanoFolder to select them. In MultiRNAFold, NUPACK and the algorithms of [16, 17], it is assumed that the RNAs are linked in a specific order. This order directly impacts on the quality of the predicted structures because the order can forbids some base pairs and so the set of all possible secondary structures is not explored. A solution could be to test all possible RNA linking orders (*n*! for *n* RNAs) but it will not guarantee that all the possible structures can be found and the number of orders to test can become high in practice.

In some RNA secondary structures, specific motifs called *pseudoknots* can occur. Pseudoknots are notably involved in the readthrough mechanism of the translation. Therefore, they are important to study the function of RNA complexes. However, pseudoknots are difficult to predict, their prediction often leading to algorithms with high execution times. Then, the prediction of RNA secondary structures with pseudoknots is often restricted to subclasses of pseudoknots [20]. When pseudoknots occur in the interaction of two RNAs, they are called external pseudoknots or crossing interactions. Among the state of the art, only NanoFolder and the algorithms of [16] are able to predict pseudoknots.

All the tools and algorithms presented above are based on different thermodynamic models aiming to minimize the free energy. However, it is now known that the real structure of an RNA is not always the structure of minimum free energy but often a structure close to it. Hence, being able to generate sub-optimal structures is an important feature for the RNA complex prediction problem. Moreover, RNAs can have several structures, as the riboswitches, forming complexes with other RNAs [21] or other molecules to regulate the gene expression. Sub-optimal structures are, especially, used together with SHAPE data to elaborate a conformational ensemble [22] that helps to determine the different states of RNAs or RNA complexes. Sub-optimal structures are also used to identify homologous ncRNAs in bacteria [23]. To our knowledge, only NUPACK provides sub-optimal structures.

Finally, among the state of the art, only NanoFolder, the NUPACK package and MultiRNAFold are available. NanoFolder is available on a web server and the NUPACK package as well as MultiRNAFold are available as sources.

Here, we propose an original approach and a tool for RNA complex prediction including pseudoknots and crossing interactions. Our approach takes advantage of the numerous tools dedicated to RNA secondary structure prediction as well as RNA-RNA interaction prediction. Indeed, an RNA complex can be considered as a set of structured RNAs interacting with each other, where the secondary structure of each RNA can impact the interactions and vice versa. For each RNA and for each pair of RNAs, several possible secondary structures can be predicted. The prediction of an RNA complex can therefore be viewed as the best combination among those different predictions that achieves the minimum free energy. Thus, given a set of RNAs, our method takes as inputs several secondary structures per RNA and several interaction sites per pair of RNAs. Then it returns several possible complexes composed of some of the inputs. The secondary structure of a single RNA, including or not pseudoknots, can be obtained by several tools, which can return sub-optimal solutions [6, 15, 24–26]. There also exist many tools to predict interaction sites between two RNAs. They do not predict crossing interactions but some can return several solutions [6, 27–29].

In this paper, we show that the RNA complex prediction problem can be defined as a combinatorial optimization problem on a graph. The possible secondary structures of each RNA and the possible interactions between each pair of RNAs are the vertices of the graph. Each vertex has a weight equals to the minimum free energy of its corresponding secondary structure or interaction. If some secondary structures and interactions can form a complex, they are said to be *compatible*. This compatibility relation between the inputs is represented with the edges of the graph. The RNA complexes we are looking for can be viewed as the combinations, with the minimum free energies, of the various inputs. Hence, the problem consists in finding the minimum weight subgraph where all the inputs are compatible with each others. This kind of subgraph, called a *clique* or a *complete graph*, is a graph in which all vertices are linked to each other. More precisely, the prediction of RNA complexes corresponds to a constrained version of the well known Maximum Weight Clique Problem (MWCP). Since solving the MWCP is NP-hard [30], several heuristics have been proposed to find good solutions in polynomial time [31]. We propose an heuristic based on Breakout Local Search [32] to find good solutions to our constrained MWCP. This heuristic allowed us to develop a tool, called RCPred, for RNA Complex Prediction. We show that compared to NanoFolder, NUPACK and MultiRNAFold, RCPred gives better results for a large set of RNA complexes.

The paper is organized as follows: we first present how the RNA complex problem can be modeled as a constrained maximum weight clique problem. Then we present the heuristic we propose to solve this problem and finally present and discuss the results we obtain with RCPred.

## Methods

As stated before, the RNA complex prediction problem can be viewed as a graph problem in which we must find a constrained clique. In this section, we first describe the relationship between the RNA complex prediction and the Maximum Weight Clique Problem (MWCP). Then, we propose an heuristic to find good solutions in a polynomial time for the constrained MWCP.

### Predicting RNA complexes: a constrained MWCP

#### RNA complexes

**Fig. 1:**
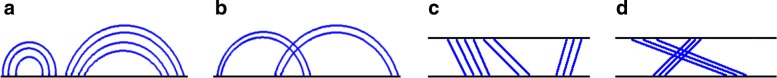
Secondary structure RNA motifs. **a** Pseudoknot-free secondary structure. **b** Pseudoknotted secondary structure. **c** RNA-RNA Interaction. **d** Crossing interaction (or external pseudoknot)

An RNA complex is composed of a set of structured RNAs that interact with each other. It can therefore be considered as a set of RNA secondary structures and of RNA-RNA interactions. A *secondary structure* involves exactly one RNA strand and is composed of a list of base pairs internal to this RNA strand (Fig. 1a). A secondary structure can contain pseudoknots (Fig. 1b). An *interaction site* (Fig. 1c) involves two RNA strands, is composed of a list of base pairs, and does not contain crossing interactions (Fig. 1d).

#### Constrained MWCP

The RNA complex prediction problem can be formalized using a weighted graph *G*(*V*,*E*) such as:

- *V*, the vertex set, is composed of two subsets, *V*^*S*^ and *V*^*I*^, where *V*^*S*^ is the set of vertices representing the secondary structures and *V*^*I*^ is the set of vertices representing the interactions. Each vertex *v*∈*V* has a weight equals to the free energy associated to the structure or the interaction.
- *E*, the edge set, represents the compatibilities between the vertices. An edge exists if and only if two vertices are compatible. We consider that two vertices are not compatible if at least two identical nucleotides are involved in different pairings or if the two vertices are secondary structures involving the same RNA strand. These compatibility rules allow the presence of any motif in RNA complexes: pseudoknots (that can already be present in the secondary structures, which are represented by the vertices *V*^*S*^) or crossing interactions.

An RNA complex can be viewed as a complete graph, or a *clique*, where each vertex is linked to all the other vertices. This clique is constrained because, for each RNA, there must be exactly one secondary structure vertex. This brings another constraint. In some known complexes, the RNAs do not have internal base pairs, which implies to add for each RNA a vertex corresponding to a secondary structure with no base pairs. However, an RNA complex only composed of secondary structures or interactions with no base pairs at all is not an RNA complex. We call the cliques corresponding to this type of RNA complexes *weak* cliques.

The weight of a clique (constrained or not) is the sum of the weights of the vertices composing the clique. We have therefore a constrained maximum vertex weight clique problem, denoted in the sequel by constrained MWCP, where the clique i) is composed of exactly one secondary structure per RNA, ii) is not weak and iii) has a minimum free energy.

#### Free energy computation

To each secondary structure and each interaction is associated a free energy represented by the vertex weights in the graph. This energy is computed to unify the different sources of secondary structures and interactions. We use two energy models:

- The first model is the Turner model [33] (with the 2004 parameter release), which can be used for secondary structure without pseudoknots and for interactions.
- The second model is based on the sum of the stacking energies taken from the Turner model (used in [24]). This allows to compute the free energy of pseudoknotted secondary structures.

Once the free energy of each secondary structure and interaction is known, it is used as the weight of each vertex. Then, the free energy of a complex (a constrained clique) is approximated by the sum of the free energies (weights) of the secondary structures and interactions (the vertices in the clique) composing it.

### Solving the constrained MWCP

The MWCP is NP-hard. However, this problem is well studied and various methods exist to solve it. Exact methods, which find the optimal solution by optimizing the weight of the clique, are either generalization of methods for the unweight problem [34, 35] or are branch and bound algorithms [36]. Since exact methods are time consuming due to the NP-hardness nature of the problem, a lot of various heuristics have been proposed, either based on local search [37], tabu search [38], both of them [32] or other techniques [39, 40].

In this paper, we propose an adaptation of the heuristic Breakout Local Search (BLS) published in [32] that provides good solutions in a short amount of time. In the following, we first present BLS and then the heuristic we propose for our constrained MWCP that we denote by BLS-CMWCP.

#### Breakout Local Search heuristic

The BLS heuristic [32] was proposed for the MWCP and is based on both local search and tabu search.

Local search [41] is an heurisitic method to find good solutions for combinatorial optimization problems. The local search is an iterative method. It starts from an initial solution and modifies it at each step by looking in its neighborhood, i.e. a set of neighboring solutions obtained by performing small modifications (movements) on the current solution, for a better solution. When a solution cannot be improved anymore, it is called a local optimum solution.

Tabu search [42] is a metaheuristic based on local search. The main difference with the local search is that at each iteration the best solution in the neighborhood of the current solution is selected, even if it is not better than the current solution. In order to avoid cycling through previously encountered solutions, a tabu list is used.

BLS starts from a random solution and then performs alternatively two phases until the known optimal solution is found or the time limit is reached:

1. Local search: to perform a local search until a local optimum is found.
2. Perturbation: to modify greatly the local optimum solution to escape from it and explore further the search space.

In the local search phase of BLS, all the possible movements are considered and the one optimizing the most the solution weight is chosen. The available movements are either to add a vertex in the clique or to replace a vertex in the clique with one that is not in the clique. To define the movements, some definitions are needed. Let *G*(*E*,*V*) be a graph and *C* be the current clique.

- *PA* is the set of vertices that can be directly added in the clique *C*, because there exist edges between them and all the vertices of the clique *C*; *PA*={*v*:*v*∉*C*,∀*u*∈*C* ∃[*v*,*u*]∈*E*}.
- *OM* is the set of pairs of vertices (*v*,*u*) where *v* is not in the clique *C* but there exist edges between it and all the vertices of the clique *C*, except *u* which is in the clique *C*; *OM*={(*v*,*u*):*v*∉*C* and *u*∈*C*,∀*v*^′^∈*C*∖{*u*}∃[*v*,*v*^′^]∈*E* and [*v*,*u*]∉*E*}. The OM set is used to do the replacement movements.
- *OC* is the set composed of all the vertices outside *C*; *OC*={*v*:*V*∖*C*}.

The perturbation phase aims to modify the current solution to escape a local optimum. The perturbation can greatly degrade the solution, the strength depending on how many times the solution was not improved in the local search phase. The perturbation strategies are based on four movements that are performed several times: to add, replace or remove a vertex of the clique (weak perturbation) and to restart (strong perturbation). The perturbation phase uses a tabu list in order to avoid to pick up again a vertex for a movement if it was removed from the solution some iterations before. The perturbation phase is a main difference with other local search methods and allows to explore more efficiently and faster the search space.

The authors show that this heuristic provides improved results for a number of MWCP instances and that this heuristic is usable for large graphs in reasonable time. It makes this heuristic a good candidate to develop a tool for the RNA complex prediction since large sets of inputs can be used.

#### The BLS-CMWCP algorithm

The BLS method is adapted for the constrained MWCP by making some modifications to the initial clique finding phase and to the movements, in order (i) to take into account the different kinds of vertices, (ii) to take into account the constraints related to the secondary structures and (iii) to avoid the weak cliques.

**Fig. 2:**
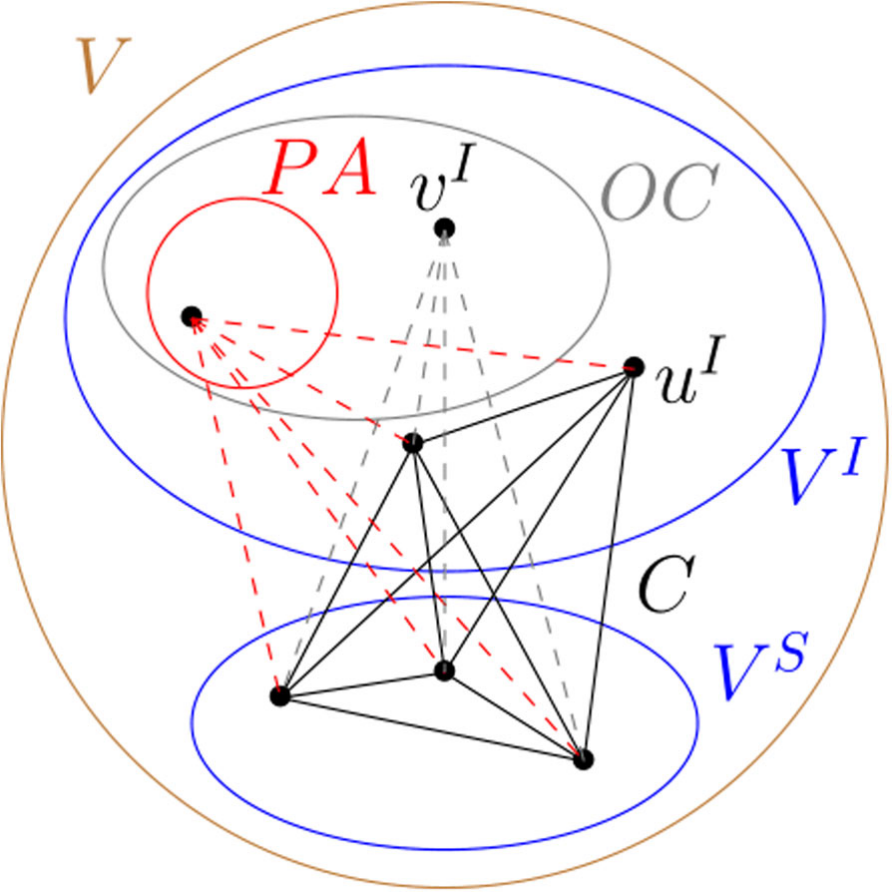
Sets used in BLS-CMWCP algorithm. *C* is a clique, *PA* is the set composed of interaction vertices that are all linked to all the vertices of the clique, *OC* is the set composed of interaction vertices that are not in the clique
- Let *OM* be the set composed of the interaction vertices pairs (*v*^*I*^,*u*^*I*^) (or secondary structure vertices pairs (*v*^*S*^,*u*^*S*^)) such that *v*^*I*^ (or *v*^*S*^) is outside *C* and is connected to all vertices in *C* except to the vertex *u*^*I*^∈*C* (or *u*^*S*^∈*C*).
- Let *OC* be the set composed of all interaction vertices that are outside *C*; *OC*={*v*^*I*^:*V*^*I*^∖*C*}.

Before describing our BLS-CMWCP algorithm, let us give the new definitions about the sets used (illustrated in Fig. 2). Let *C* be a clique from the initial graph *G*(*E*,*V*).

- Let *PA* be the set composed of all the interaction vertices that are outside *C* and are connected to all the vertices in *C*; *PA*={*v*^*I*^:*v*^*I*^∉*C*,∀*u*∈*C* ∃[*v*^*I*^,*u*]∈*E*}.

Having only the interaction vertices *v*^*I*^ in *PA* and in *OC* and having only interaction vertex pairs (*v*^*I*^,*u*^*I*^) or secondary structure vertex pairs (*v*^*S*^,*u*^*S*^) in *OM* allow only the movements respecting the constraint of having exactly one secondary structure per RNA. In the following, we describe each modification and the differences between our algorithm BLS-CMWCP and BLS:

- **To generate the initial clique:** in BLS, the phase consists in selecting randomly a vertex and then to add iteratively vertices if they form a clique, until no more vertex can be added. In BLS-CMWCP, this phase consists in selecting randomly an interaction vertex and then selecting for each RNA a secondary structure vertex that forms a clique. Forming a clique is always possible thanks to the empty secondary structures which are obviously compatible with any interaction vertex.
- **To add a vertex movement:** an interaction vertex *v*^*I*^ is selected in *PA* and added into *C*.
- **To replace a vertex movement:** a vertex pair (*v*^*I*^,*u*^*I*^)(or(*v*^*S*^,*u*^*S*^)) is selected in *OM*, *v*^*I*^ (or *v*^*S*^) is added to *C* and *u*^*I*^ (or *u*^*S*^) is removed from *C*.
- **To remove a vertex movement:** an interaction vertex *v*^*I*^ is selected in *C* to be removed.
- **To restart the clique movement:** an interaction vertex *v*^*I*^ from *OC* is added to the clique *C*. Then if the structure vertices of the clique, *v*^*S*^∈*C*, do not form a clique, they are replaced with other structure vertices. Finally, the remaining interaction vertices of the clique, *v*^*I*^∈*C*, are removed if they do not form a clique anymore.
- **To generate sub-optimal cliques:** contrary to BLS method, we want to return several sub-optimal cliques. In BLS-CMWCP, at each iteration of the local search and of the perturbation phases, any new clique is saved. Then when the solutions are returned, they are sorted according to their free energy.
- **To forbid the weak cliques:** during all the search, if a movement leads to a weak clique, it is not considered.

#### RCPred: implementation of BLS-CMWCP for RNA complex prediction

The BLS heuristic proposed in [32] has a set of parameters to modulate the strength of the perturbation (*L*_0_ and *L*_*Max*_), the maximum number of non-improving solutions before a strong perturbation is performed (*T*), the coefficients for accepting non-improving solutions (*α*_*s*_ and *α*_*r*_), the coefficient for tabu tenure (*ϕ*) and the probability for applying directed perturbations (*P*_0_). Some parameters were fixed in the BLS heuristic and we used them as such in BLS-CMWCP. We determined the other parameters by performing experiments on a dataset of 30 graphs derived from RNA complexes. We then chose the following parameters: *L*_0_=0.1∗|*V*|, *L*_*Max*_=0.1∗|*V*|, *T*=10, *α*_*s*_=0.5, *α*_*r*_=0.5, *ϕ*=7 and *P*_0_=1. The stop condition for the local search occurs either when the optimum clique is found or the maximum number of iterations is reached. In RNA complex prediction we do not know the optimum clique, then the stop condition here is a maximum number of iterations (fixed at 500). This parameter can be set by the user.

We implemented in C++ BLS-CMWCP and obtained the tool called RCPred (RNA Complex Prediction). RCPred takes as inputs *n* sequences of RNAs, several secondary structures per RNA and several interactions per pair of RNAs. First the energies of the secondary structures and the interactions are computed. The compatibilities between the secondary structures and the interactions are determined and the graph is built. BLS-CMWCP returns constrained cliques from which are derived RNA complexes. If some sequences are identical, symmetrical complexes can occur. These lasts are identified and removed to avoid redundancies in the results. Finally, the RNA complexes are sorted according to their free energy. RCPred is available on the EvryRNA platform.

## Results

In this section, we present the results we have obtained with RCPred on a large set of RNA complexes. First we detail below the dataset used and how we recovered the secondary structures and interactions. We then give the results of RCPred and compare it with NanoFolder [19], NUPACK [15] and MultiRNAFold [13].

### Datasets and RCPred inputs

In the following experiments, we use a dataset composed of 90 non-redundant RNA complexes. The dataset is extracted from the database RNA STRAND [43] that gathers 4,666 secondary structures of single and multi-strand RNAs. All the recovered complexes are experimentally validated by NMR or X-ray and are not composed of modified nucleotides. We are interested here in complexes longer than 20 nucleotides and smaller than 1000 nucleotides. Because of the complexity in time of the tools from the state of the art, we exclude the complexes longer than 1000 nucleotides in order to be able to generate several secondary structures and interactions. The dataset of the benchmark is available on the EvryRNA platform.

For each RNA, we generated the secondary structures using three tools from the literature for RNA secondary structure prediction, namely BiokoP [24], pKiss [26] and RNAsubopt (from the ViennaRNA package [6]). We chose these three tools because they are able to generate sub-optimal solutions. Moreover, they have good performances as shown in [24]. For each of the three tools, we fixed the maximum number of sub-optimal solutions to 30. Note that BiokoP and pKiss can predict pseudoknots while RNAsubopt predicts only pseudoknot-free secondary structures. We merged the results of the three tools to have a diversified set of secondary structures for each RNA. To generate the interactions between each pair of RNAs, we used the tool RNAsubopt [6] which can also predict RNA-RNA interaction sites with numerous sub-optimal solutions. We fixed the maximum number of sub-optimal solutions to 90.

### Prediction results

We present in this section the results obtained by our tool RCPred and the comparison with the results of NanoFolder, NUPACK and MultiRNAFold. As RCPred is based on an heuristic, the results presented here are obtained from 5 executions. Among these tools, only RCPred and NUPACK can return sub-optimal solutions corresponding to different predicted complexes.

#### Statistics used

To evaluate the quality of predicted complexes, we used the sensitivity to measure the ability of finding positive base pairs and the Positive Predictive Value (PPV) to measure the ability of not finding false positive base pairs. We also used the F_1_-score which is the harmonic mean between the sensitivity and the PPV and the Mathews Correlation Coefficient (MCC) which is a balanced measure between sensitivity and specificity (that measures the proportion of negatives that are correctly identified). These statistics are computed as follows:

$$Sensitivity=\frac{TP}{TP+FN}, \text{} {PPV}=\frac{TP}{TP+FP} $$

$$\mathrm{F}_{1}\text{-score}=2 \times \frac{Sensitivity \times {PPV}}{Sensitivity + {PPV}} $$

$${{}\begin{aligned} {MCC}=\frac{TP \times TN - FP \times FN}{\sqrt{(TP+FP) \times (TP + FN) \times (TN + FP) \times (TN + FN)}} \end{aligned}} $$

where *TP* is the number of true positive base pairs, *FN* is the number of false negative base pairs, *FP* is the number of false positive base pairs and *TN* the number of true negative base pairs.

#### RCPred evaluation

##### Sub-optimal solutions

**Fig. 3:**
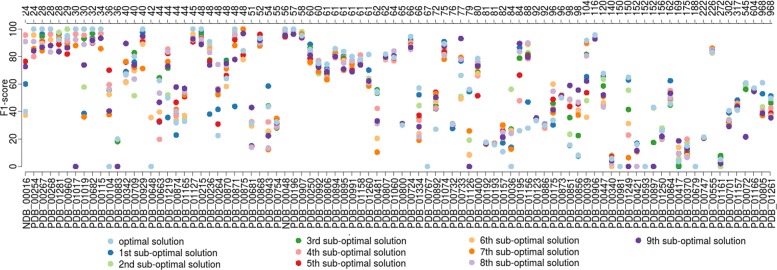
F_1_-score results of RCPred. Results are shown for the 10 first solutions returned by RCPred in average on 5 executions on our dataset. The secondary structure inputs are generated by BiokoP, pKiss and RNAsubopt and the interaction inputs are generated by RNAsubopt. The complex lengths are indicated in nucleotides in the superior axis

We first study for each complex the optimal and sub-optimal predictions of RCPred in order to see the relevance of generating several solutions. We report on Fig. 3 the F_1_-score results of the 10 first solutions returned by RCPred in average on 5. As we can see, the solutions having the highest F_1_-scores are in most cases the first returned. However, in many cases, the sub-optimal solutions reach the highest F_1_-scores, there are even some complexes for which the best prediction is given by the tenth sub-optimal solution. This confirms that better predictions can be found in the sub-optimal solutions and therefore the need to generate them.

##### Influence of the inputs

**Fig. 4:**
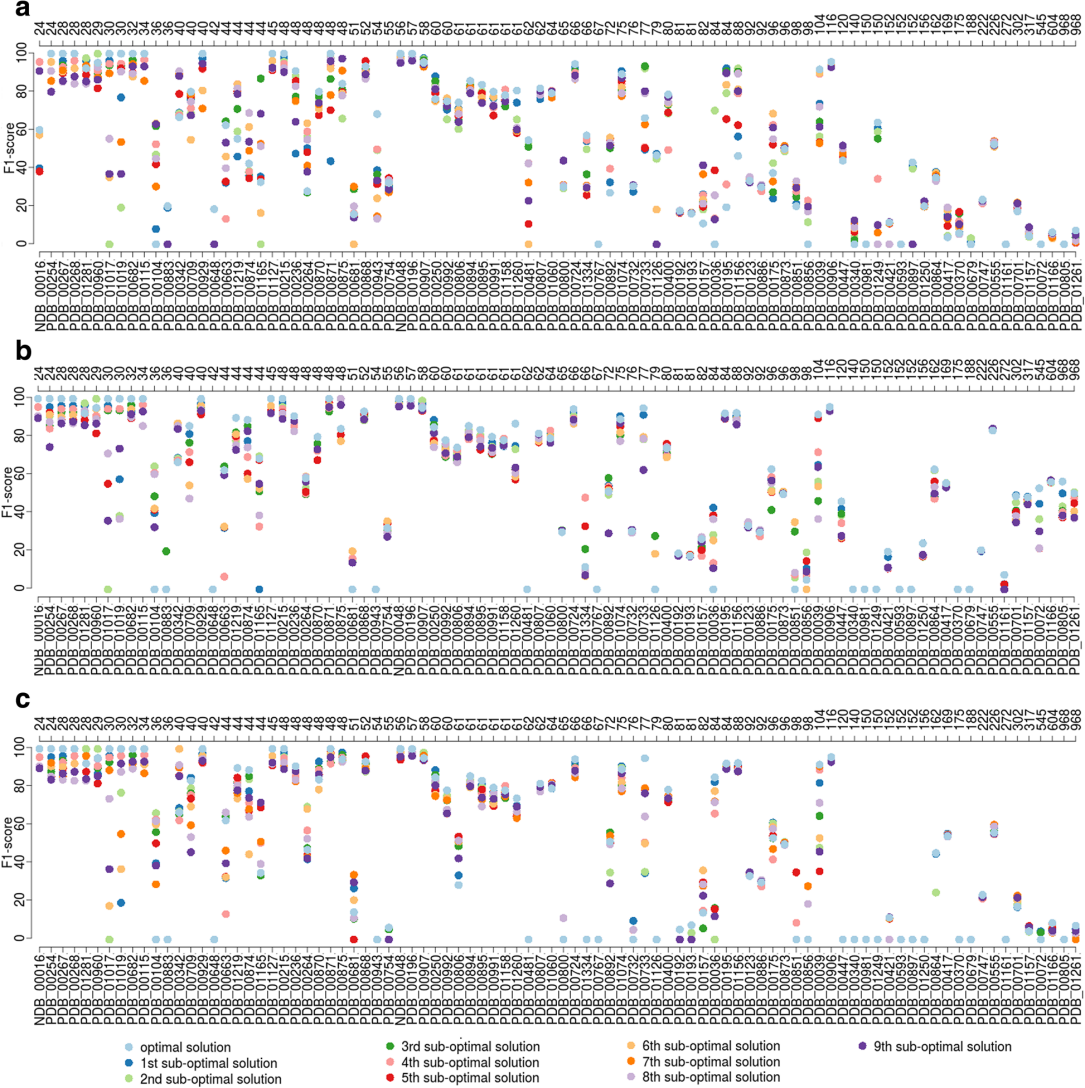
F_1_-score results of RCPred in function of the inputs. Results are shown for the 10 first solutions returned by RCPred in average on 5 executions on our dataset. **a** The secondary structure inputs are generated by BiokoP. **b** The secondary structure inputs are generated by pKiss. **c** The secondary structure inputs are generated by RNAsubopt.The complex lengths are indicated in nucleotides in the superior axis

We study here the influence of the inputs by taking either the secondary structures generated by BiokoP, pKiss or RNAsubopt alone. We report on Fig. 4 the F_1_-score results of the 10 first solutions returned by RCPred in average on 5 executions, with inputs of BiokoP (A), pKiss (B) and RNAsubopt (C). Knowing that the performances of BiokoP, pKiss and RNAsubopt depends on the size of the RNAs, it is expected that they all predict accurate secondary structures for small RNAs. Hence it is not surprising to observe similar results for the smallest complexes (inferior to 62 nucleotides). For longer complexes the results differ. A comparison of these results with the ones obtained when the input secondary structures generated by the three tools are merged (Fig. 3) shows that we obtain better results when the inputs are merged. This strongly suggests that when the inputs are merged, the best secondary structure inputs are selected by RCPred. This confirms the first interest of RCPred, which is to deal with numerous and varied possible secondary structures and interactions, in order to predict efficiently and in low time computing RNA complexes.

#### Comparison with the state of the art

**Fig. 5:**
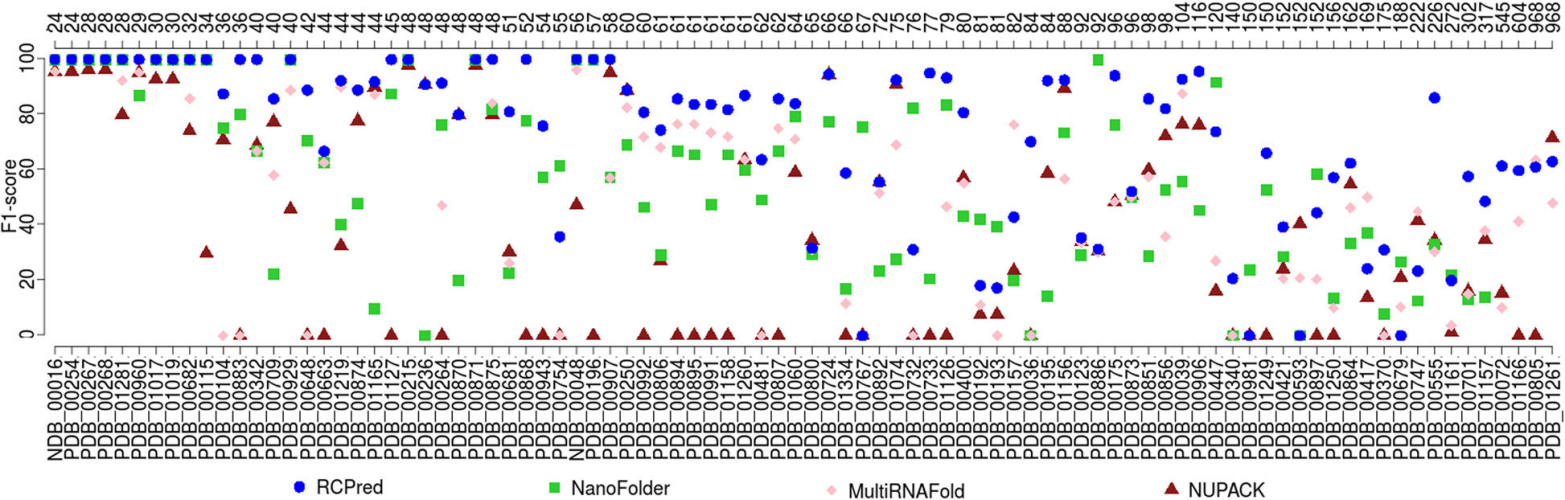
F_1_-score results of RCPred, NanoFolder, NUPACK and MultiRNAFold. For RCPred and NUPACK, the maximum F_1_-scores among the 10 first returned solutions are taken for each complex to compute the mean. For RCPred, the mean is computed over 5 executions. The complex lengths are indicated in nucleotides in the superior axis

We then compare RCPred with NanoFolder, NUPACK and MultiRNAFold. We report the F_1_-score results of these tools on our dataset in Fig. 5. We execute RCPred 5 times and, for each execution, we recover the maximum F_1_-score solution of the 10 first solutions returned for each complex. We then compute the mean of these recovered solutions. For NUPACK, we report the maximum F_1_-scores among the 10 first solutions returned. Note that we could not test NanoFolder (which is usable through a web server) on the longest complexes (of size greater than 550 nucleotides), because of the size limitation of the web server. Also we encounter some difficulties with the tool MultiRNAFold on some complexes.

As we can see on Fig. 5, RCPred obtains better F_1_-scores than the other tools in most cases. With the sub-optimal solutions, we can guarantee that at least a predicted structure is close to the referenced one. For almost all complexes smaller than 120 nucleotides, RCPred is able to find accurate predictions with F_1_-scores higher than 80%. For longer complexes, RCPred, as well as the other tools, becomes less effective with a maximum F_1_-score around 70%. Similar results and findings are obtained with sensitivity, PPV, and MCC statistics (see Additional file 1). We summarize these results by reporting the average on all the dataset in Table 1. As NanoFolder and MultiRNAFold were not able to give results for some complexes, their means are done only on the complexes that were successfully predicted. The results show that in average RCPred is able to predict complexes more accurately than NanoFolder, NUPACK and MultiRNAFold.

**Table 1:** Mean sensitivity, PPV, F_1_-score and MCC results of RCPred, NanoFolder, NUPACK and MultiRNAFold on our dataset

	RCPred	NanoFolder	NUPACK	MultiRNAFold
Sensitivity	**65.8**	61.9	37.9	54.6
PPV	**70.5**	50.4	41.6	56.0
F_1_score	**67.3**	54.9	39.2	54.8
MCC	**67.5**	55.0	38.8	54.6

For RCPred and NUPACK, the maximum F_1_-scores among the 10 first returned solutions are taken for each complex to compute the mean. The corresponding means of sensitivity, PPV and MCC are given. For RCPred, the mean is computed over 5 executions. Bold text indicates the higher scores

## Conclusion and perspectives

In this paper, we propose a new method and a tool, called RCPred, to predict secondary structures of RNA complexes composed of several RNAs.

We model the problem of RNA complex prediction with input knowledge as a constrained maximum weight clique problem in a graph and we present an heuristic based on Breakout Local Search to find good solutions, resulting in the tool RCPred. This modeling allows to predict all kinds of RNA complex motifs including pseudoknots or crossing interactions. This is not the case of the tools NUPACK and MultiRNAFold that do not predict at all these motifs.

RCPred is also able to provide sub-optimal solutions. Generating sub-optimal solutions is very important in RNA secondary structure prediction for several reasons. First, because it is known that the real structure is not always the structure of minimum free energy but often a structure close to the structure of minimum free energy. Moreover, a model cannot capture all the subtleties of the minimum free energy computation of an RNA complex of more than two strands. Sub-optimal solutions are then needed to allow to cover the discrepancies of the model. Among the existing tools, only the tool from the NUPACK package can generate sub-optimal solutions.

We test RCPred on a large dataset composed of 90 RNA complexes of various lengths, including or not pseudoknots. We show that RCPred is able to predict accurately RNA complex secondary structures and gives competitive results compared to NanoFolder, NUPACK and MultiRNAFold. Each returned RNA complex has a global free energy resulting from the sum of the free energies of the secondary structures and of the interactions composing it. A perspective could be to improve the global free energy computation of the complexes (by adapting for example the calculation method used in RNAeval from ViennaRNA package [6]) and reorder them accordingly.

The time execution of RCPred varies between 0.05 s in average on 5 executions for the smallest complex and 16.7 seconds for the longest. We are currently working on optimizing the time execution.

In RNA complexes, the tertiary interactions are numerous and have an important role in the stabilization of the global structure. There exist databases gathering 3D motifs appearing in single RNA structures, like the Rna3Dmotif database [44]. Moreover, it has been shown that inserting 3D motifs in RNA secondary structures helps in improving the prediction [45]. A perspective for this project would be to insert 3D motifs of single RNAs and of interacting RNAs in the predicted RNA complex secondary structures.

## Additional file

Additional file 1 Supplementary statistical results. Supplementary Figures showing the sensitivity, PPV and MCC results of RCPred compared to the results of NanoFolder, NUPACK and MultiRNAFold. (PDF 61.8 kb)

## Abbreviations

BLS: Breakout local search
BLS-CMWCP: Breakout local search-constrained maximum weight clique problem
BLS-MWCP: Breakout local search-maximum weight clique problem
MWCP: Maximum weight clique problem
