# Supplementary material for: RCPred: RNA complex prediction as a constrained maximum weight clique problem
Source: BMC Bioinformatics. 2019 Mar 29;20(Suppl 3):128. doi: 10.1186/s12859-019-2648-1 (PMC6439972; doi:10.1186/s12859-019-2648-1)
Supplement: Supplementary file 1 — Supplementary statistical results. Supplementary Figures showing the sensitivity, PPV and MCC results of RCPred compared to the results of NanoFolder, NUPACK and MultiRNAFold. (PDF 61.8 kb) [file 12859_2019_2648_MOESM1_ESM.pdf]

RESEARCH

Additional File of “RCPred: RNA Complex Prediction as a constrained maximum weight clique problem”

Audrey Legendre, Eric Angel and Fariza Tahi*

*Correspondence:
fariza.tahi@univ-evry.fr
IBISC, Univ Evry, Université Paris-Saclay, 91025 Evry, France
Full list of author information is available at the end of the article

Additional results

Figure 1 Sensitivity results of RCPred, NanoFolder, NUPACK and MultiRNAFold. For RCPred and NUPACK, the maximum F_1-scores among the 10 first returned solutions are taken for each complex to compute the mean. The mean sensitivity of these solutions is then computed. For RCPred, the mean is computed over 5 executions. The complex lengths are indicated in nucleotides in the superior axis.

Figure 2 PPV results of RCPred, NanoFolder, NUPACK and MultiRNAFold. For RCPred and NUPACK, the maximum F_1-scores among the 10 first returned solutions are taken for each complex to compute the mean. The mean PPV of these solutions is then computed. For RCPred, the mean is computed over 5 executions. The complex lengths are indicated in nucleotides in the superior axis.

Figure 3 MCC results of RCPred, NanoFolder, NUPACK and MultiRNAFold. For RCPred and NUPACK, the maximum F_1-scores among the 10 first returned solutions are taken for each complex to compute the mean. The mean MCC of these solutions is then computed. For RCPred, the mean is computed over 5 executions. The complex lengths are indicated in nucleotides in the superior axis.
